# Radiomics Diagnostic Tool Based on Deep Learning for Colposcopy Image Classification

**DOI:** 10.3390/diagnostics12071694

**Published:** 2022-07-12

**Authors:** Yuliana Jiménez Gaona, Darwin Castillo Malla, Bernardo Vega Crespo, María José Vicuña, Vivian Alejandra Neira, Santiago Dávila, Veronique Verhoeven

**Affiliations:** 1 Departamento de Química y Ciencias Exactas, Universidad Técnica Particular de Loja, San Cayetano Alto s/n, Loja CP1101608, Ecuador; dpcastillo@utpl.edu.ec; 2 Instituto de Instrumentacion para la Imagen Molecular I3M, Universitat Politécnica de Valencia, E-46022 Valencia, Spain; 3 Theoretical and Experimental Epistemology Lab, School of Optometry and Vision Science, University of Waterloo, Waterloo, ON N2L3G1, Canada; 4 Facultad de Ciencias Médicas, Universidad de Cuenca, Cuenca 010203, Ecuador; bernardo.vegac@ucuenca.edu.ec (B.V.C.); joshevicuna@hotmail.com (M.J.V.); vivian.neira@ucuenca.edu.ec (V.A.N.); santiagoadavilas@gmail.com (S.D.); 5 Family Medicine and Population Health, University of Antwerp, 2610 Antwerp, Belgium; veronique.verhoeven@uantwerpen.be

**Keywords:** cervical coloscopy, deep learning, Unet, lesion classification

## Abstract

Background: Colposcopy imaging is widely used to diagnose, treat and follow-up on premalignant and malignant lesions in the vulva, vagina, and cervix. Thus, deep learning algorithms are being used widely in cervical cancer diagnosis tools. In this study, we developed and preliminarily validated a model based on the Unet network plus SVM to classify cervical lesions on colposcopy images. **Methodology:** Two sets of images were used: the Intel & Mobile ODT Cervical Cancer Screening public dataset, and a private dataset from a public hospital in Ecuador during a routine colposcopy, after the application of acetic acid and lugol. For the latter, the corresponding clinical information was collected, specifically cytology on the PAP smear and the screening of human papillomavirus testing, prior to colposcopy. The lesions of the cervix or regions of interest were segmented and classified by the Unet and the SVM model, respectively. **Results:** The CAD system was evaluated for the ability to predict the risk of cervical cancer. The lesion segmentation metric results indicate a DICE of 50%, a precision of 65%, and an accuracy of 80%. The classification results’ sensitivity, specificity, and accuracy were 70%, 48.8%, and 58%, respectively. Randomly, 20 images were selected and sent to 13 expert colposcopists for a statistical comparison between visual evaluation experts and the CAD tool (*p*-value of 0.597). **Conclusion:** The CAD system needs to improve but could be acceptable in an environment where women have limited access to clinicians for the diagnosis, follow-up, and treatment of cervical cancer; better performance is possible through the exploration of other deep learning methods with larger datasets.

## 1. Introduction

Cervical Cancer is the second leading gynecological cancer in Latin American women [1]. The specific situation in Ecuador involves a high cancer mortality rate of cervical and stomach cancers [2]. Furthermore, increasing rates of thyroid, breast, lung, and colorectal cancer were reported in women, while in men the incidences remained stable. In 2020, 1534 new cases were detected, and 813 women died of this cause. The incidence of cervical cancer in Ecuador has reached 17.8/100,000 women. The number of yearly deaths has increased in Ecuador over the last 10 years, and has therefore attracted the interest of public policies [3,4,5].

Vaccination against Human Papillomavirus (HPV) infection and cervical cancer screening for the early detection and treatment of cervical intraepithelial neoplasia are effective strategies for cervical cancer prevention [1]. According to the severity of the cervical precancerous lesions, they can be divided into three types of cervical intraepithelial neoplasms (CIN I, CIN II, and CIN III). Thus, colposcopy is used to evaluate abnormal or indecisive cervical cancer screening tests. Visual inspection is performed after the application of acetic acid to highlight precancerous or cancerous abnormalities [1,4].

In 2020, the World Health Organization (WHO) launched the strategy 90-70-90 to eradicate cervical cancer worldwide. This initiative recommends that 90% of the population should be vaccinated against HPV, 70% of the population should be screened with a high-sensitivity test at least twice during their lifetime (at 35 and 45 years), and 90% of women with cervical abnormalities should be followed up. Vaccination, screening, and follow-up need to be improved in low- and middle-income countries (LMIC). However, the applicability of these treatments to women in less developed countries remains largely untested; especially in Ecuador, vaccination is unpopular and a registry is absent [6]. Screening is performed, but at least 40% of women are never screened in their lifetime, and the follow-up of screening is greatly compromised by the lack of capacity and dropouts because of long waiting times, the availability of a colposcope, and the availability of trained personnel. Morbidity and mortality mainly hit women with vulnerable socioeconomic status, ethnic minorities, and women in rural areas (geographically isolated)—risk circumstances that are often connected.

Likewise, the problem with colposcopy is that the accurate prediction of malignant lesions depends on the experience of the gynecologist, who is required to be able to identify and deal with changes in the acetic acid white epithelium according to standards. With the emergence of computer intelligence technologies and deep learning algorithms, deep convolutional neural networks (CNNs) especially have become one of the most useful architectures in the medical imaging process, and have achieved good performance results in segmentation and classification tasks [7,8,9]. Thus, a Computer-Aided Diagnosis (CAD) system is helping as a second opinion to detect anomalies and malignant tumors in medical images.

The aim of this study is to present an alternative method for cervical examination with colpophotography, and to compare the colposcopy diagnosis with CAD colposcopy.

## 2. Related Work

Several authors have developed deep learning architectures to assist in the early diagnosis of cervical cancer. Yuang et al. [7] built a Unet model for cervix lesion segmentation and Mask R-CNN 43 for the detection model. The average accuracy of the U-Net model in acetic images was 95.59%, its recall was 84.73%, and its DICE was 61.64%. The colposcopy images were input to a pre-trained multi-modal (acetic and iodine images) ResNet classification model, achieving a sensitivity, specificity, and accuracy of 85.38%, 82.62% and 84.10%, respectively, and an AUC of 0.93. Lui et al. [2] proposed a CAD system for colposcopy image classification using a ResNet model. The positive and negative classification results indicate that ResNet and clinical features perform better than ResNet alone. The AUC, accuracy, sensitivity, and specificity were 0.953, 0.886, 0.932, 0.846, 0.838, and 0.936, respectively.

Likewise, Chandran et al. [8] implemented two CNN models—(i) VGGNet19 and (ii) Colposcopy Ensemble Network (CYENET)—to classify cervical cancers from colposcopy images automatically. They demonstrated that the CYENET model is superior for the classification with an accuracy of 92.3%, which is 19% higher than the VGG19’s 73.3%. Cho et al. [9] developed CNN (InceptionResnetv2 and Resnet152) models to automatically classify cervical neoplasms on colposcopy images with an AUC of 0.947 ± 0.030 by Resnet152. Zhang et al. [10] proposed a CAD method for the automatic classification of HSIL (high-grade intraepithelial lesion) or higher-level lesions in colposcopy images based on transfer learning and pre-trained densely connected CNN. This method achieved an accuracy of 73.08% over 600 test images.

In the same way, Miyagi et al. [11] built a CNN with 11 layers, and showed a high accuracy of 82.3%, a sensitivity of 80%, and a specificity of 88.2% for the classification of LSIL and HSIL+. Sato et al. [12] developed a CNN using, as input, a total of 445 colposcopy images classified into three groups: severe dysplasia, carcinoma in situ, and invasive cancer. The accuracy using data augmentation on the validation dataset was ~50%.

In this project, a CAD tool is proposed for automatic colposcopy image segmentation and classification tasks, aiming to reduce the workload of specialists in colposcopy diagnosis and improve detection accuracy.

## 3. Materials and Methods

**Figure 1:**
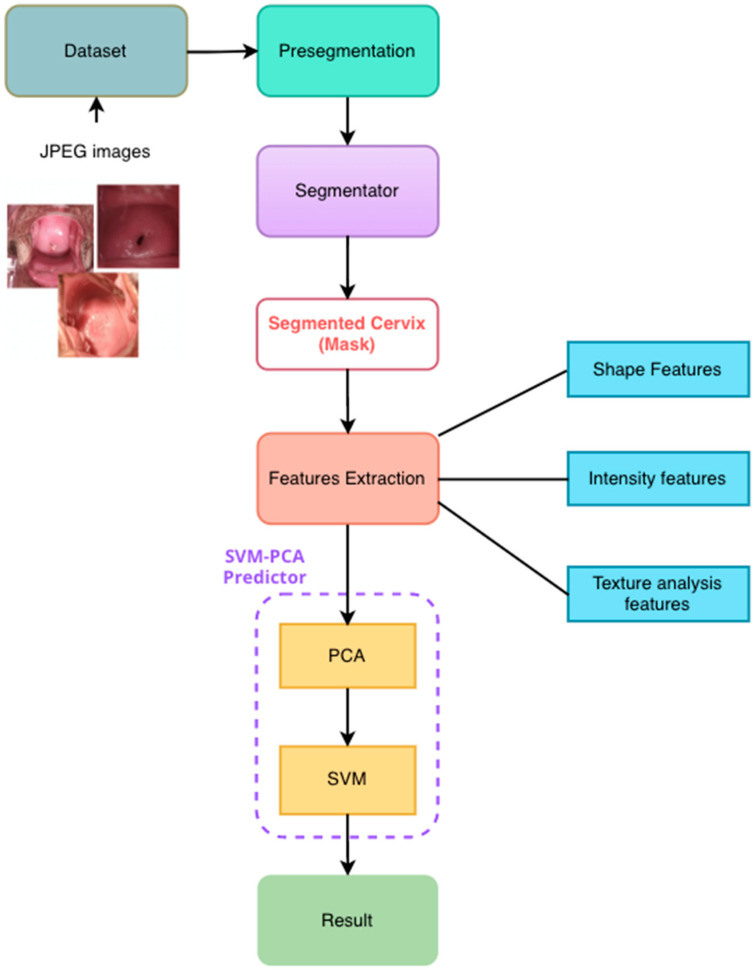
Computer-Aided Diagnostic tool flowchart in cervix images. First, manually cropped regions of interest (ROIs) were extracted. Then, synthetic images were generated by data augmentation from the colposcopy RoIs. After that, synthetic and real data were used as inputs for the training of the Unet model and to solve the segmentation problem. Then, some features were extracted from the ROIs, and SVM (Support Vector Machine Learning) was implemented as a classification method. Finally, evaluation metrics were used to evaluate the performance of the models.

Figure 1 describes the general workflow of the deep learning and machine learning methods for ROI segmentation and classification, respectively.

### 3.1. Datasets

The colposcopy databases used in this project were:

(i) The public database “Intel & Mobile ODT Cervical Cancer Screening” from Kaggle community developers https://www.kaggle.com/competitions/intel-mobileodt-cervical-cancer-screening/data (accessed on 1 October 2021). It contains 1481 cervix images divided into two categories based on their visual aspect: normal (specified as “considered non-cancerous”) and abnormal. From these, 460 were taken as a training set in this work.
(ii) A private dataset was collected from women from a rural community in Ecuador. All of the images were anonymized and collected from June to December 2020, as a part of the project CAMIE—https://www.camieproject.com/ (accessed on 2 June 2022). Table 1 shows the classification of two image datasets according to their negative or positive diagnosis of lesions.

### 3.2. Private Data Collection

The private dataset was composed of images collected from 64 participants of the CAMIE project, who tested positive for HPV or had presented an abnormal pap smear and were referred to colposcopy. During the colposcopy examination after the acetic acid application, the cervix visualizations were classified as normal or abnormal by a reference colposcopist, who had the information of the HPV test and pap smear prior to his colposcopy evaluation, as is standard practice [13]. This classification is considered to be the golden standard for the present study.

After classification, images of the cervix were taken. The device selected for taking all of the images was a cellphone (Xiaomi company Redmi Note 9), which was previously tested against other devices to select the best one. The colpophotographs were taken at 4 cm from the vaginal speculum using a zoom of 4× without flash. All of the colpophotographs were anonymized and stored in a private repository, and a total of 64 colpophotographs were collected.

From those, 20 images were selected to be analyzed with a CAD system as testing data. The selection of the images was based on their quality, with good illumination and the cervix centered in the picture. These pictures were then evaluated by 13 expert colposcopists, and their assessment was compared with the result of the tool. All of the statistical data were processed using IBM SPSS v 25.

### 3.3. Data Augmentation

The number of medical images provided by the databases was still insufficient for the training of the Unet model and the avoidance of the overfitting problem. In order to solve this problem, data augmentation [14,15] techniques were used to obtain an improvement in model performance.

**Figure 2:**
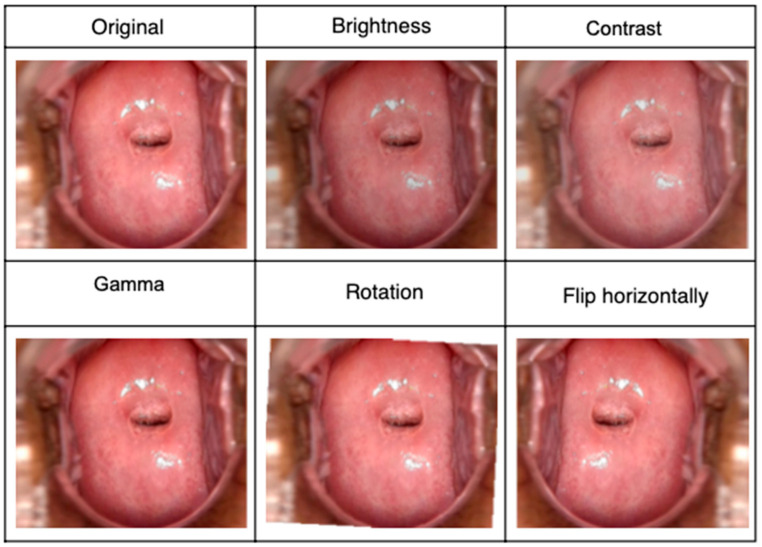
Some original images and the geometric transformations which were used in the data augmentation.

The public collection of images (Figure 2) was increased using basic data augmentation techniques. Specifically, the following transformations were used: contrast, brightness, gamma, rotation, and flipping the image horizontally [16].

A total of 460 images were used as the CNN input using the cross-validation technique: 368 as the training set (80%) and 92 as the validation set (20%).

### 3.4. Presegmentation: Cropping the Regions of Interest (RoI)

**Figure 3:**
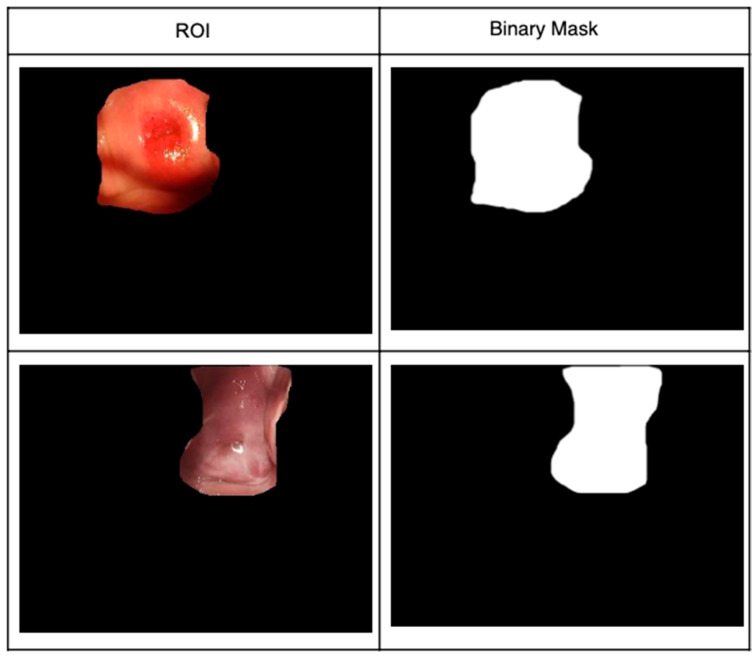
Manual RoI selections and their masks.

The Region of Interest (ROI) is the region that allows the identification, measurement, and evaluation of the risk of cervical cancer. First, all of the ROIs in the images were selected and annotated manually by expert physicians using the ImageJ software (Figure 3). Then, masks were used by the Unet neural network as a semiautomatic method of ROI segmentation.

In the pre-segmentation blocks (see Figure 3), all of the images were cropped in the central zone, where the lesions (ROIs) to be analyzed are shown.

### 3.5. Segmentation

**Figure 4:**
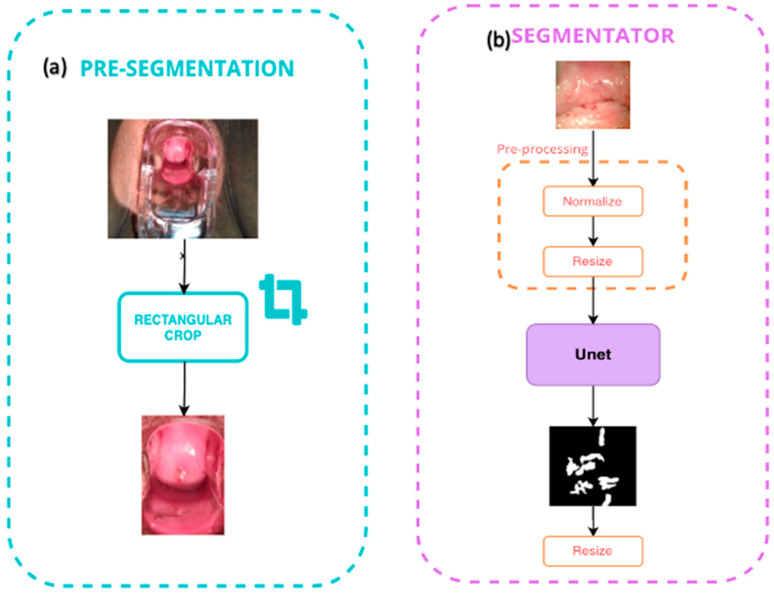
(**a**) Pre-segmentation block; (**b**) segmentation block.

In the segmentation, all of the preprocessed images were normalized and resized from 512 × 512 to 128 × 128 pixels to feed the Unet network to obtain a model with the capability to segment the ROIs from the images. See Figure 4b.

The U-net architecture consists of two main paths, down sampling and up sampling, and the model was inspired by the original work described in [16]. The hyperparameter training details used in Unet are described in Table 2, and were implemented using python libraries such as TensorFlow, Keras, scikit–learn and Google Colab, using a total of 460 images, where 80% (368) were used for training and 20% (92) were used for validation.

### 3.6. Features of the Extraction and Classification

After the ROI cervix images’ segmentation, it is important to extract the principal features to differentiate the lesions, which will be classified as normal or abnormal.

In this project, a total of 92 radiomic features were extracted from each region of interest (ROIs) through pyradiomics. These features were divided into first-order statistics (like entropy and energy), a gray level co-occurrence matrix (GLCM), a gray level run length matrix (GLRLM), a gray level size zone matrix (GLSZM), a gray level dependence matrix (GLDM), and neighboring gray-tone difference matrix (NGTDM). The reader is referred to these references [17,18,19] in order to obtain more details from algorithms and descriptions about them.

After the extraction of the features, the Principal Component Analysis (PCA) was used as a method of feature selection with the aim to reduce the redundancy, interference, and noise of the features vector. In that way, the features will be the more useful information to use in the classification–prediction algorithm.

The classification of the normal and abnormal images was achieved using the Support Vector Machine (SVM) algorithm. The fivefold cross-validation was performed using the test set to evaluate model performance.

The SVM method was introduced by Boser et al. [20] as one of the pattern recognition methods. The SVM classifies between two classes by constructing a hyperplane in high-dimensional feature space. More information and details on SVMs are given in the literature [21].

## 4. Results

The most important results, assessment metrics, and graphs obtained from the Unet and SVM training are discussed in this section.

### 4.1. Cervix Image Segmentation with Unet

**Figure 5:**
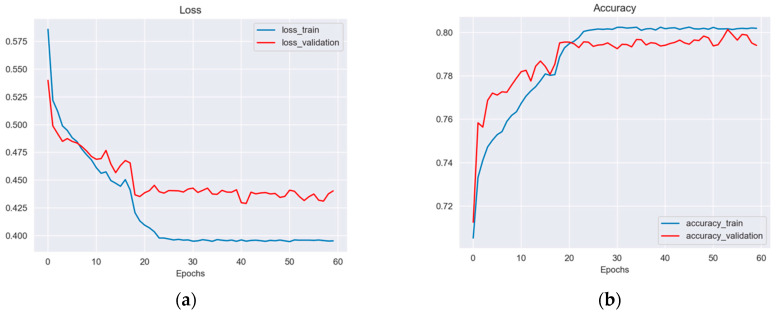

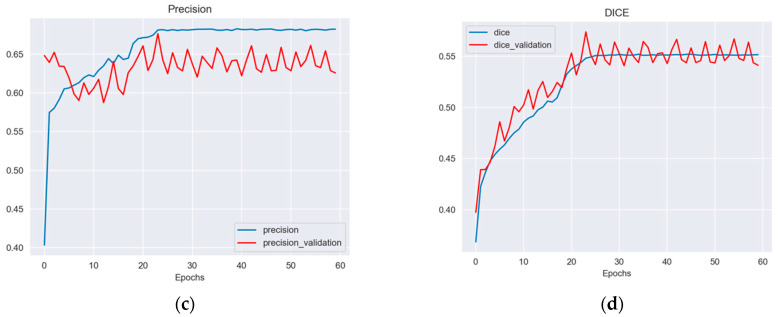
The plotting graphs show several metrics ((**a**) Loss, (**b**) accuracy, (**c**) precision, and (**d**) DICE) concerning the different number of epochs during the training and validation of the Unet architecture.

Figure 5a–d shows the training and validation models using Unet architecture. The figures show the plot indices obtained for each epoch during the testing model, such as the loss value, accuracy, precision, and DICE concerning each epoch in the Unet model. The results demonstrate that Unet is a more stable network, with a better constant learning rate and less overfitting.

### 4.2. Cervix Image Classification with SVM

The features allow the implementation of the SVM-PCA classifier used in this work, in order to determine the probability (prediction) of the image fed into the CAD system resulting in an image with a risk of cancer.

**Figure 6:**
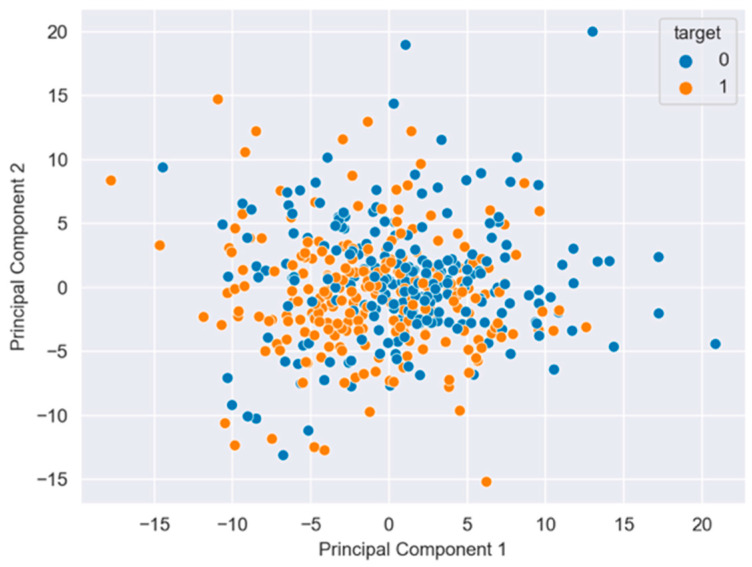
The PCA analysis shows the dispersion and independence between two 0 and 1 classes.

Figure 6 summarizes the information from the features extracted by PCA and the confusion matrix. The features allow the implementation of the SVM classifier to determine the probability (prediction) of the image fed into the CAD system resulting in an image with or without a risk of cancer. The results are an accuracy of 58%, a sensitivity of 70%, and a specificity of 48.8%.

Despite the fact that the Unet method demonstrated good segmentation results (an accuracy of 80%), the classifier based on SVM requires some improvement related to the accuracy (58%). Thus, the Unet + SVM model doesn’t outperform other similar works presented in the literature using deep learning [8,22] or machine learning [23,24,25,26] models. Hence, as a future work, we intend to evaluate deep convolutional neural networks such as Resnet or Densenet as cervix image classification models, looking to improve the accuracy results.

### 4.3. Graphical Interface

**Figure 7:**
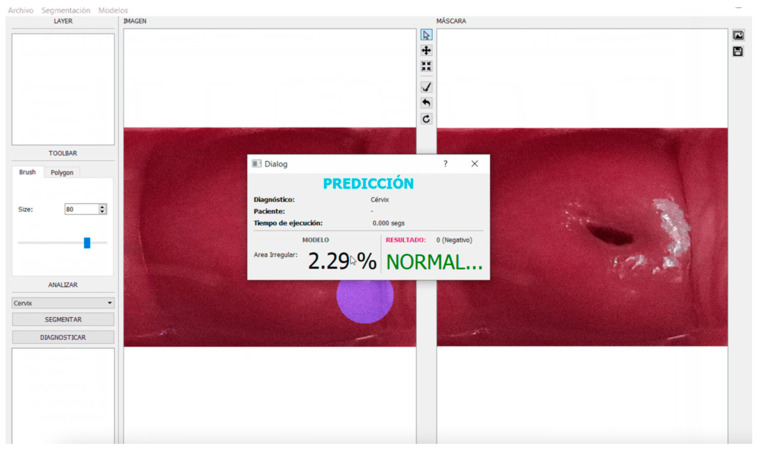
Graphical Interface for colposcopy image classification.

Finally, a graphical interface was developed using the PyQt libraries, which can segment and classify benign and malignant lesions. The tool can be found for free at the following link: https://user-images.githubusercontent.com/15198470/141326567-829ba3f0-d64b-43c4-8fc7-38febfce32dd.gif (accessed on 2 June 2022); see Figure 7.

### 4.4. Visual Comparison

Additionally, we present a preliminary comparison of the CAD system results [27,28] with the results taken from 13 colposcopy experts (Table 3). The hypotheses are: (i) H_0_, the results between visual experts’ evaluations and the CAD tool are similar, and (ii) H_1_, the results between visual experts’ evaluations and CAD tool are non-similar. The *p*-value of 0.597 indicates no statistically significant difference between the experts and the CAD tool for the interpretation of the images.

## 5. Discussion

The ROI segmentation reached the following scores: an Intersection over Union (IoU) of 0.5, an accuracy of 0.80, a precision of 0.65 and a DICE of 0.55 across all of the ROI augmented datasets, demonstrating that our UNet model obtained similar results with a low number of images in comparison with other CNN models [16,29].

The metrics are not like another models [30,31,32] where the IoU index overcomes our results (Figure 5a–d). For example, in [7] a Unet network was used for colposcopy image classification based on a Resnet model, with an IoU value of more than 0.5. This could be due to the number of images used in their work (2233), which allowed them to improve their performance percentages in the segmentation and classification task.

The U-Net was shown to be the most appropriate for this task because it is normally the most useful model in biological image segmentation. Recent literature agrees with this last proposition, e.g., Liu et al. [30] compared U-Net, FCN and SegNet models as an image segmentation method for cervical squamous intraepithelial lesions, the results indicated that Unet outperformance the other two models with the following scores: average pixel accuracy (MPA) of 0.8736, the average intersection (MIoU) of 0.7786, and the frequency weight intersection (FWIoU) of 0.7895. In the same way, Liu et al. [31] reported Unet as colposcopy image segmentation where the experimental results showed that the proposed model has a better effect on colposcopy cervical image segmentation.

This model has also been used in similar works [7,22,33,34] as a combination of the DL method as segmentation and then as an input of the ML method as classification. Elayaraja et al. [23] used machine learning methods for cervical lesion segmentation and classification: the system performances reached were 97.42% sensitivity, 99.36% specificity, 98.29% accuracy, PPV 97.28%, an NPV of 92.17%, an LRP of 141.71, an LRN of 0.0936, 97.38% precision, 96.72% FPR and 91.36% NPR. Zhang et al. [22] proposed a Unet model for cervix image segmentation and a CapsNet model for classification; the training set accuracy was 99%, and the test set accuracy was 80.1%.

Our results were compared with other DL [21,35,36] and ML methodologies [24,25] with the principal aim to obtain the best metric values referring to the classification of cervical cancer through the images. For example, Liang et al. [35] developed a method using SVM with different kernels and low-level features (color, edge and texture boundary features), for which they reported a success classification rate of 94.6%. However, they were only training the SMV with 48 and 40 sets of positive and negative cancer lesion images, respectively. Meanwhile, Thohir et al. [37] used the GLCM feature extraction and the SVM classifier with the polynomial kernel to identify cervical cancer, and reported an accuracy of 90%, the data used was composed of 500 images.

In this sense, Park et al. [38] compared some machine learning and deep learning methods for the classification of cervical cancer based on images. They reported that the ResNet-50 model showed a 0.15-point improvement (0.97) over the average (0.82) of the three machine learning methods (SVM, XGB, and RF), SVM obtained 0.84, the features collected in the entire dataset were 300, and they selected only 10.

In this project, the metric results were a sensitivity of 70%, a specificity of 48.8%, and an accuracy of 58%. Although these values are inferior to the last work mentioned [8], it is important to indicate that, like that project, we established a comparison of the results with expert colposcopists (Table 3), where they deduced that their system performed better in ordinary colposcopy images than in high-definition images, and in the case of ours, the comparison is good but susceptible to improvement when the training data will be larger.

Therefore, it is important to explore the combination of the DL and ML methods in order to obtain the best results; currently, a common proposal in other areas of medical image classification reported successful results—e.g., Ragab et al. [26]—through the deep convolutional neural network architecture, and the SVM classifier like the last layer of this architecture (DCNN-based SVM) obtained an AUC of 0.94. In our case, we propose to use of a U-Net architecture with an SVM-PCA, the latter one is used to reduce the principal features of the ROIs and in that way optimize the computational time.

Thus, our results demonstrate that the sensitivity of colposcopy images, on average, is acceptable when the image is read by a colposcopist. However, there is great variation in the performance of individual colposcopists, and similar findings are reported by Yuan et al. [7]. As colposcopy and colpophotography are operator-dependent results, several internal or external factors such as the expertise and time of the interpretation could affect the result.

Whereas real-time colposcopy allows a dynamic view of the cervix and a 3-D visualisation, a system with static colpophotograpy and computer-aided diagnosis with sensitivity and accuracy could be an acceptable screening instrument in an environment where women have limited access, or no access at all, to expert colposcopists. The diagnosis of machine learning could be improved according to the number of images available; another advantage is that the inter-operator variation will be lower.

## 6. Conclusions

Our results indicate that synthetic images improve the colposcopy diagnosis performance of our AI tool for the classification of cervix images. We propose a combined Deep Learning method (Unet) plus Machine Learning (SVM) to obtain the best index of identification, classification, and prediction of cervix abnormalities through the processing of images. However, the problem of limited data in this project motivated us to apply methods for synthetic data augmentation in order to enlarge the colposcopy dataset.

For the reason mentioned above, basic geometric data augmentation techniques were used to generate new synthetic data. However, affine geometric techniques normally used in object data augmentation, are less adequate for medical data augmentation because these methods only generate synthetic images with a similar distribution to the original ones, causing data overfitting. Thus, deep learning models that are recently being used in data augmentation and segmentation for the training of convolutional neural networks are generative models. Generative Adversarial Networks (GANs) have recently become the most useful in generating more realistic images, achieving good results in medical synthetic image generation, because they use the latent space to evaluate the model, generating new realistic images based on the data distribution. These models introduce the problem of non-convergence and a diminishing gradient; these significantly improve the performance of segmentation tasks.

Likewise, deep learning training models such as convolutional neural networks (CNNs) based on GANs need more data with realistic distributions.

In future work, we aim to collect more private normal and abnormal cervix images in order to improve the performance of deep learning models. Our purpose will be to implement GAN models for data augmentation and segmentation tasks, and to improve training classification using CNN models, looking to avoid ineffectual treatments and unnecessary biopsies.

Finally, if our model proves to be accurate, its utilization potential is considerable, especially in low-resource settings with limited specialized health personnel.

## Figures and Tables

**Table 1:** Summary of the datasets used in this project.

Datasets	Real Images		
	Negative	Positive	Total
Intel & Mobile ODT Cervical Cancer Screening (public)	130	130	360
CAMIE (private)	6	14	20
Data augmentation	50	50	100
Total	236	244	480

**Table 2:** The U-net training hyperparameter details.

Hyperparameters	Unet
Number of epochs	200
Batch size	3
Steps	123
Steps validation	30
Optimizer	Adam
Learning rate	0.0005
Loss validation	0.63
Loss function	Binary-cross entropy
Activation function	ReLu, Sigmoid

**Table 3:** Statistical comparison between the visual evaluation experts and the CAD tool.

	Mean Paired Samples	95% Confidence Interval	*p* Value
Colpo-Experts	70	48–92	0.597
Neural Network	71	54–96	

## Data Availability

Links for the reported results and private dataset analyzed during the study: https://www.camieproject.com (accessed on 2 June 2022); https://github.com/Hikki12/colpografia-app (accessed on 2 June 2022); https://drive.google.com/drive/folders/1Am7R2Ty0WRuCUHmPB9ypZ-n8Vr-LTDeg?usp=sharing (accessed on 2 June 2022). The visitor needs to register to obtain an ID before being allowed into the database, and please credit the VLIR project as the source of the data at the end of your paper if you use this data in a publication.

## Abbreviations

AUC	area under the curve
CC	cervical cancer
CNN	convolutional neural network
CAMIE	Cáncer Auto Muestreo Igualdad Empoderamiento
CIN	cervical intraepithelial neoplasms
CAD	computer-aided diagnosis/detection
DL	deep learning
FCN	full connected layer
GAN	generative adversarial network
HPV	human papillomavirus
IoU	intersection over union
PPV	positive predictive value
PCA	principal component analysis
NPV	negative predictive values
RoI	region of interest
SVM	support vector machine
DICE	similarity index of two images/samples
SIL	squamous epithelial lesions
VLIR-UOS	Vlaamse Interuniversitaire Raad Universitaire Ontwikkelingssamenwerking (Flemish Interuniversities Council University Development Co-operation)
WHO	World Health Organization
